# Clinical exome sequencing by general pediatricians: high clinical utility and no evidence of inappropriate testing

**DOI:** 10.3389/fped.2024.1392444

**Published:** 2024-04-22

**Authors:** Danya Salah Baz, Dareen Baz, Fawzah Alrwuili, Abdullah Aldowaish, Hanan E. Shamseldin, Ayman Elhomoudi, Fowzan S. Alkuraya

**Affiliations:** ^1^ College of Medicine, Alfaisal University, Riyadh, Saudi Arabia; ^2^ Department of Pediatrics, King Faisal Specialist Hospital and Research Center, Riyadh, Saudi Arabia; ^3^ Department of Translational Genomics, Center for Genomic Medicine, King Faisal Specialist Hospital and Research Center, Riyadh, Saudi Arabia; ^4^ Department of Pediatrics, King Faisal Specialist Hospital and Research Center, Madinah, Saudi Arabia

**Keywords:** exome sequencing (ES), General Pediatrics, clinical utility, autosomal recessive diseases, genetic disorders

## Abstract

**Background:**

Genetic disorders account for a large percentage of admissions and outpatient visits to children's hospitals around the world. Clinical exome sequencing (CES) is a valuable diagnostic tool in the workup of these disorders; however, it is not routinely requested by general pediatricians. This may represent a missed opportunity to increase patient access to this powerful diagnostic tool. In our institution, general pediatricians can directly order CES. In this context, this study aims to evaluate the appropriateness of CES and its clinical utility when ordered by general pediatricians.

**Methods:**

We retrospectively reviewed all CES tests ordered by general pediatricians in our institution between 2019 and 2023 and recorded their indications and results. General pediatricians were interviewed to evaluate how CES impacted the domains of clinical utility by assessing changes in management, communication, subsequent testing, and counseling. In addition, feedback was obtained, and barriers faced by general pediatricians to order CES were assessed.

**Results:**

The study cohort (*n* = 30) included children from the inpatient (60%) and outpatient (40%) departments. A positive finding (a pathogenic or likely pathogenic variant that explains the phenotype) was observed in 11 of 30 cases (37%), while 3 (10%) and 16 (53%) received ambiguous (variant of uncertain significance) and negative results, respectively. The indication was deemed appropriate in all 30 cases (100%). Clinical utility was reported in all 11 positive cases (100%). Reproductive counseling is a notable utility in this highly consanguineous population, as all variants identified, in the 11 positive cases, were autosomal recessive.

**Conclusion:**

We show that CES ordered by general pediatricians is appropriately indicated and provides a diagnostic yield comparable to that requested by specialists. In addition, we note the high clinical utility of positive results as judged by the ordering pediatricians. The findings of this study can empower general pediatricians to advocate for expanded CES adoption to improve patient access and shorten their diagnostic odyssey.

## Background

Genetic diseases notably impact pediatric healthcare systems worldwide. The advent of diagnostic genomics, specifically clinical exome sequencing (CES) and clinical genome sequencing (CGS), has revolutionized the field of genetic diagnostics by significantly shortening the time to diagnosis for patients with rare diseases. However, the utilization of these powerful tools in clinical practice remains limited, often restricted to specialized geneticists or other subspecialists because of hospital regulations and concerns about clinical utility.

Despite general pediatricians being on the frontlines of managing most genetic diseases, there is a lack of data on the utilization of CES and CGS by them. This is particularly striking when considering the routine use of karyotyping, a genomic test that general pediatricians are comfortable ordering. Unlike karyotyping, CES has a much higher diagnostic yield, especially when it includes copy number analysis (1). Since CES is more complex and relatively new compared with karyotyping, ordering CES often requires referral to geneticists. This common practice, however, can severely restrict patient access to this powerful diagnostic tool, especially when one considers the shortage of clinical genetics professionals (2–4). On the other hand, empirical data are needed on how well CES is utilized by general pediatricians before advising wider adoption. The King Faisal Specialist Hospital and Research Center (KFSH&RC) in Saudi Arabia, has implemented an inclusive approach that allows general pediatricians to request CES when suspecting a genetic etiology. This presented us with an opportunity to investigate this experience and explore the diagnostic yield and clinical utility of CES when requested by general pediatricians. The findings of this research have the potential to inform the debate on how best to promote access of patients with genetic diseases to the latest diagnostic tools without compromising the quality of healthcare utilization.

## Methods

### Cohort selection

The KFSH&RC is a tertiary healthcare facility in Saudi Arabia. The General Pediatrics service, in Riyadh, accepts acute and chronic pediatric patients as well as complex cases from different regions in Saudi Arabia and covers 4,715 outpatients and 617 inpatients annually. On average, children are admitted for seven days for diagnostic evaluations and treatment plans. In this study, we reviewed all exome orders submitted through our clinical laboratory to identify those ordered by members of the General Pediatrics team between 2019 and 2023 for patients suspected of harboring a genetic etiology of their diseases. Each family received an extensive explanation of CES analysis, including the benefits and drawbacks of the test, with an emphasis on secondary findings followed by the signing of a written informed consent form. The physician gathered the medical histories of the probands and their families and created an annotated pedigree. Afterward, blood samples were obtained from the proband and, where applicable, from other family members, and used for genetic testing. This study was conducted following the tenets of the Declaration of Helsinki and with the approval of the Institutional Review Board at the KFSH&RC (RAC#2230016).

### Clinical utility

Baseline characteristics were recorded for each patient upon enrollment, including current age, gender, phenotype at presentation, and parental consanguinity. Physicians were surveyed to gather their input on the clinical utility of WES, utilizing the four domains developed by Dimmock et al. (5) and adopted by Monies et al. (6):

Category 1: Major perceived specific changes in acute patient management or clinical outcome: These include screening for potential comorbidities associated with the genetic disease diagnosis, new subspecialty consulted, changes in medications, changes in invasive procedures (including decisions regarding transplant and termination), changes in diet, changes in imaging studies, and changes in palliative care. Changes in clinical outcome were assessed by the successful use of targeted treatments, avoidance of complications, and institution of palliative care.
Category 2: Changes in communication: These include communication with families regarding outcomes, expectations, and prognosis.
Category 3: Changes in subsequent test ordering, that is, triggering additional confirmatory tests (testing for comorbidities was not included here because it was a part of category 1).
Category 4: Changes in other types of care (counseling, further monitoring, or research studies).

A “Yes” answer to any of the above domains was recorded as “positive” to count instances where a result had positive clinical utility.

### Exome sequencing and bioinformatics analysis

CES was performed on the NovaSeq platform using the following protocol: Exons were captured and enriched using Illumina DNA Prep with Enrichment. Enrichment-bead-linked transposons (eBLTs) were used to tagment 100–500 ng of gDNA and attach adapter sequences to the fragments. After eBLT cleanup, two indices per sample by PCR amplification (five cycles) were added. Subsequently, individual libraries were pooled for a single hybridization reaction and capture. The last step consisted of a postcapture PCR amplification (eight cycles) prior to sequencing on a NovaSeq 6000 sequencer (Illumina, Inc., USA) as 150 bp paired-end reads, following the manufacturer's protocols (Illumina, Inc., USA). The DNA sequence was mapped and analyzed in comparison with the published human genome build (UCSC hg19 reference sequence) using a local installation of the Illumina DRAGEN Server v3 20040619 pipeline. Variants were reported as “pathogenic,” “likely pathogenic,” and “uncertain significance” according to the ACMG variant interpretation guidelines (7). When no variants that potentially explained the clinical indication were identified, the case was reported as negative. Reports of pathogenic or likely pathogenic variants that fully explained the clinical indication and in the right zygosity were considered positive. Those with variants of uncertain significance (VUS) were considered ambiguous. Secondary findings were reported according to the latest ACMG list of secondary findings (8).

### Genetic result reporting and follow-up

Families were invited for a follow-up visit or phone call with the primary physician after receiving the findings of the CES, during which recommendations and changes to clinical management were recorded, and further referrals for additional consults or diagnostic tests were made as applicable. In addition, they were referred to genetic counselors to discuss the results, understand the possibility of recurrence in subsequent pregnancies, and inform them of their reproductive options.

## Results

### Patients and phenotypes

A total of 30 patients were recruited for this study, as summarized in Table 1. The gender of the patients was predominantly male (60%), and consanguinity was reported in 60% of families. The most common phenotype in our cohort involved the gastrointestinal system (60%). In addition, dysmorphic features were observed in 30% of all cases. CES revealed a genetic diagnosis in 11 out of 30 children (36.7%) of the cohort (Table 2). Three out of 30 patients (10%) reported ambiguous results, requiring further investigation. Notably, patients with a positive CES result had higher rates of gastrointestinal involvement (81.8%) compared with the other phenotypes (Table 3). All 11 patients with a positive result displayed an autosomal recessive pattern of inheritance. Nine out of these 11 were from consanguineous families.

**Table 1:** Demographic and phenotypic characteristics of the 30 probands evaluated by whole exome sequencing (WES).

Characteristics	Value (%)
Gender	
Male	18 (60.0%)
Female	12 (40.0%)
Age (years)	
Mean	5.7
Range	0.3–17
Primary phenotype at presentation^a^	
Gastrointestinal (including failure to thrive)	18 (60.0%)
Developmental delay	12 (40.0%)
Facial dysmorphism	9 (30.0%)
Musculoskeletal/integumentary	7 (23.3%)
Cardiovascular	5 (16.7%)
Immunology	5 (16.7%)
Respiratory	5 (16.7%)
Neurological	4 (13.3%)
Nephrology and urology	3 (10.0%)
Hematology and coagulation	2 (6.7%)
Endocrine	2 (6.7%)
Ophthalmology	1 (3.3%)
CES result	
Positive	11 (36.7%)
Negative	16 (53.3%)
Ambiguous	3 (10%)
Total	30

^a^ Each patient may present with more than one phenotype.

**Table 2:** Cases with positive molecular findings.

Patient No.	Sample tag	Phenotype	Clinical Utility	Variant	Genotype	OMIM-compatible Diagnosis
1	CGM2022-01326-1	Congenital diarrhea with hypochloremia	Prognostication (life-long disease), management (diagnosis-specific treatment), reproductive counseling (25% recurrence risk)	*SLC26A3*: NM_000111.3:c.559G>T:p.(Gly187*)	Homozygous	Congenital secretory chloride diarrhea 1 (OMIM# 214700)
2	MDL2016-00594-4	Blistering, skin fragility, autism, hypothyroidism, multidysplastic left kidney, delayed development in speech and cognition (Figure 1C).	Management (supportive), reproductive counseling (25% recurrence risk)	*FERMT1*: NM_017671:c.676dupC:p.(Gln226Profs*17)	Homozygous	Kindler syndrome (OMIM# 173650)
3	CGM2022-03141-2	Dysmorphic features, kyphoscoliosis, restrictive respiratory insufficiency, moderate to severe hypotonia, global developmental delay	Prognostication (life-long disease), management (supportive), reproductive counseling (25% recurrence risk)	*STAC3*: NM_001286257.2:c.293G>C:p.(Trp98Ser)	Homozygous	Congenital myopathy 13 (OMIM# 255995)
4	MDL2019-00575-3	Immunodeficiency, failure to thrive, chronic diarrhea	Prognostication (life-long disease), management (disease-specific treatment), reproductive counseling (25% recurrence risk)	*RIPK1*: NM_003804:c.1934C>T:p.(Thr645Met)	Homozygous	Immunodeficiency 57 with autoinflammation (OMIM# 618108)
5	CGM2023-01698-2	Dysmorphic features, hypotonia	Management (supportive), reproductive counseling (25% recurrence risk)	*MYH2*: NM_001100112.1: c.4537+1G>A	Homozygous	Congenital myopathy 6 with ophthalmoplegia (OMIM# 605637)
6	CGM2022-00150-1	Chronic diarrhea, failure to thrive, hypothyroidism	Management (TPN until small bowel transplant is done), reproductive counseling [complex based on recurrence of one or two diseases as described in (9)]	*EPCAM*: NM_002354.2: c.38T>C: p.(Leu13Pro) *BEST1*: NM_004183.4:c.197_198del:p.(Leu67Valfs*164)	Homozygous	Tufting enteropathy with congenital diarrhea 5 (OMIM# 613217) and bestrophinopathy, autosomal recessive (OMIM# 611809)
7	MDL2019-02281-1	Chronic diarrhea, failure to thrive, hypothyroidism	Management (TPN until small bowel transplant is done), reproductive counseling (25% recurrence risk)	*EPCAM*: NM_002354:c.499dupC:p.(Gln167Profs*21)	Homozygous	Tufting enteropathy with congenital diarrhea 5 (OMIM# 613217)
8	CGM2022-03289-2	Chronic diarrhea, failure to thrive, hepatosplenomegaly, anemia	Management (chemotherapy, bone marrow transplant), reproductive counseling (25% recurrence risk)	*STXBP2*: NM_001127396.3:c.1421C>T:p.(Pro474Leu)	Homozygous	Familial hemophagocytic lymphohistiocytosis 5, with or without microvillus inclusion disease (OMIM# 613101)
9	MDLREQ2020-0352	Vascular malformation, mandibular hyperostosis, recurrent gingival bleeding, myopia (Figure 1B)	Management (conservative), reproductive counseling (25% recurrence risk)	*ELMO2*: NM_133171.3: c.1802-1G>C	Homozygous	Primary intraosseous vascular malformation (OMIM# 606893)
10	CGM2023-02023-1	Dysmorphic features, obesity, global developmental delay, xanthomas (Figure 1A)	Prognostication (life-long disease), management (diagnosis-specific treatment), reproductive counseling [complex based on recurrence of one or two diseases as described in (10)]	*MAN1B1*: NM_016219.5: c.2072A>G:p.(His691Arg) *LDLR*: NM_000527.5:c.2027del:p.(Gly676Alafs*33)	Homozygous	Rafiq syndrome (OMIM# 614202) and familial hypercholesterolemia 1 (OMIM# 143890)
11	CGM2023-02483-1	Seizures, spasticity, muscle weakness, hypokalemia, hypocalcemia, hypomagnesemia	Prognostication (life-long disease), management (diagnosis-specific treatment), reproductive counseling (25% recurrence risk)	*SLC12A3*: NM_000339.3:c.247C>T:p.(Arg83Trp)	Homozygous	Gitelman syndrome (OMIM# 263800)

**Table 3:** Disease categories of the 11 positive probands evaluated by CES.

Positive phenotypes	No. (%)
Gastrointestinal (including failure to thrive)	9 (81.8%)
Musculoskeletal/integumentary	4 (36.4%)
Facial dysmorphism	3 (27.3%)
Developmental delay	2 (18.2%)
Immunology	2 (18.2%)
Neurological	2 (18.2%)
Hematology/vascular	2 (18.2%)
Endocrine	2 (18.2%)
Respiratory	1 (9.1%)
Nephrology	1 (9.1%)
Ophthalmology	1 (9.1%)

Each patient may present with more than one phenotype.

### Clinical utility of CES ordered by general pediatricians

The CES results proved highly beneficial with one or more aspects of clinical utility in all the 11 positive cases (100%). These results are displayed in Table 4.

(1) Major perceived specific changes in acute patient management

**Table 4:** Numerical breakdown of clinical utility (based on the four domains described in the text).

Changes in acute patient management or clinical outcome	*n* = 9^a^
Changes in clinical outcome or prognosis	9
Changes in medications	7
Changes in diet	5
Changes in imaging studies	5
Changes in invasive procedures	4
Resulted in screening for comorbidities	5
Changes in communication	*n* = 11^a^
Communicates a specific diagnostic label not suspected prior to WES	6
Explains the natural history of disease	11
Changes in subsequent test ordering	*n* = 6^a^
Changes in other types of care	*n* = 9
Counseling	9
Further monitoring	3

^a^ Please note that there is an overlap in the cases and corresponding categories.

CES enabled targeted therapy in seven of 11 children (63.6%), while supportive management was continued in the remaining 4 children (36.4%). Of the seven children, four stood out as the CES result helped determine the patients’ eligibility for a transplant as follows:

Patient 4 (Table 2) presented as a 9-month-old with chronic diarrhea since the age of 1 month. His past medical history was notable for colitis, multiple scalp abscesses, gluteal abscess, and fistulation. CES resulted in a diagnosis of *RIPK1*-related Immunodeficiency-57 with autoinflammation (OMIM# 618108). Elucidating the molecular diagnosis allowed for appropriate management, as the patient underwent an allogenic stem cell transplant.

Patient 6 (Table 2) presented as an 11-month-old with chronic diarrhea and severe failure to thrive starting at the age of 2 weeks. Her past medical history was notable for hypothyroidism, diagnosed at the age of 1 month. A skeletal survey showed severe osteopenia. CES resulted in a diagnosis of *EPCAM*-related tufting enteropathy (OMIM# 613217). Elucidating the molecular diagnosis allowed for appropriate management, as the patient was placed on total parenteral nutrition (TPN) and is enlisted to receive a small bowel transplant.

Patient 7 (Table 2) presented as a two-month-old with severe failure to thrive, chronic diarrhea, and history of recurrent hypoglycemia and treated hypothyroidism. The abdominal ultrasound showed a mildly increased echogenicity of the liver with no focal lesion, a distended gallbladder, and dilated fluid-filled bowel loops with increased peristalsis. The skeletal survey showed generalized osteopenia associated with wasting of the muscles of the trunk, upper limbs, and lower limbs bilaterally, suggestive of disuse atrophy. CES resulted in a diagnosis of *EPCAM*-related tufting enteropathy (OMIM# 613217). Elucidating the molecular diagnosis allowed for appropriate management, as the patient was placed on TPN and is enlisted to receive a small bowel transplant (similar to patient 6).

Patient 8 (Table 2) presented as a 2-month-old with failure to thrive, diarrhea, and fever. She experienced generalized edema with severe dehydration that required PICU admission for 28 days. She was transferred to the regular inpatient floor where she stayed for an additional month because of hepatomegaly, ascites, and elevated liver enzymes. CES resulted in a diagnosis of *STXBP2*-related Familial hemophagocytic lymphohistiocytosis (OMIM# 613101). Elucidating the molecular diagnosis allowed for appropriate management, as she was started on chemotherapy and received an allogenic stem cell transplant.

(2) Changes in communication within healthcare teams and with families

In 54.5% of the cases (6/11), general pediatricians appreciated being able to communicate diagnoses not suspected before CES. They explained the natural history of the disease to the family while offering an informed prognosis.

(3) Changes in subsequent test ordering

The molecular diagnosis triggered subsequent test ordering in 6 of the 11 positive cases (54.5%); however, screening for comorbidities and other features of the disease were omitted as they were a part of category 1.

(4) Changes in other types of care (counseling, further monitoring, or research studies)

The autosomal recessive nature of the causal variant in all positive cases (100%) enabled reproductive counseling for the patient families. Parents were referred to genetic counselors for counseling, in which they were offered options for preimplantation genetic testing or prenatal diagnosis for future pregnancies as well as cascade carrier testing to identify relatives at risk. Patient 10 had two positive primary findings that revealed homozygosity for *MAN1B1*: NM_016219.5:c.2072A > G: p.(His691Arg) and *LDLR*: NM_000527.5:c.2027del:p.(Gly676Alafs*33). She had a strong family history of hypercholesterolemia that was consistent with the *LDLR* variant. CES was also ordered to help diagnose the patient's neurodevelopmental disorder, and a diagnosis of Rafiq syndrome (OMIM# 614202) was made by using the identified *MAN1B1* variant.

Similarly, recurrence risk was complicated in Patient 6 who was found to have in addition to the primary finding of *EPCAM*-related tufting enteropathy the unexpected finding of *BEST1*-related inherited retinal disease. The latter is known to be age-dependent so long-term monitoring of eye involvement was advised.

### Feedback on CES

General pediatricians, during the interviews, expressed their unanimous support for the use of CES. They stated that they were comfortable ordering CES for their patients and believed that it is an essential tool for diagnosing those with ambiguous clinical presentations. They also found it helpful in providing a clinical prognosis and in guiding patient management plans. They strongly advocated for the continued use of CES, because early detection of diseases can help improve the overall outcome for patients.

## Discussion

This study aimed to determine whether general pediatricians can effectively order and utilize CES to improve the diagnostic odyssey of their patients. The findings demonstrated that they can indeed effectively use CES to improve patient care because all orders were properly indicated and all 11 positive cases had positive clinical utility outcomes, demonstrating its value. This study may have the effect of persuading other institutions to empower pediatricians with the authority to order CES while more evidence is generated. This is critical because general pediatricians are usually the first to assess every child who arrives at the hospital.

Early diagnosis of genetic diseases in children is crucial. Many of these diseases manifest during childhood, making prompt diagnosis and treatment imperative. Unfortunately, medical genetics professionals who can request CES are currently in short supply (2–4). Consequently, some patients may not receive the necessary tests on time, which could lead to severe consequences. To tackle this challenge, more physicians, starting with general pediatricians, should be encouraged to order CES. The National Human Genome Research Institute (NHGRI) foresees the possibility of genomic testing becoming as routine as a complete blood count test by 2030 (11). Therefore, physicians must become more familiar with these tests to provide the best care for their patients. By diagnosing genetic diseases early and administering prompt treatment, clinicians can enhance the quality of life of children with genetic ailments.

Our study cohort included children and infants in both inpatient and outpatient settings with a diagnostic rate of 36.7% and a demonstrable clinical utility in all positive cases.

Our findings showed that early disease diagnosis resulted in better prognostication and management of patients. In most of the positive cases (54.5%, 6/11), the pediatricians had not suspected the diagnosis that was revealed by CES. One strength of our study is its inclusion of both inpatient and outpatient settings. In contrast, the study conducted by Kagan et al. (12) consisted of infants and children only in the inpatient setting. This is notable, as the relatively high diagnostic yield demonstrates a significant proportion of genetic etiologies not only among severely affected children requiring complex medical care but also among those manifesting with more common general pediatric phenotypes.

General pediatricians are typically the first clinicians to be approached by families of ill children, highlighting the importance of a high index of suspicion to the possibility of genetic etiology. Following up on this clinical suspicion with CES remains the best diagnostic course of action when compared with other ancillary diagnostic modalities. Apart from the high diagnostic yield, the results of this study reflect the real-world clinical utility of CES. It is important to note that before every CES test, a formal detailed explanation was given to the children’s families during which it was explained that there existed a possibility of secondary/incidental findings being reported. It was explained that these findings may include information on genes that are associated with other diseases and that the families have the right to decide whether they want more information on this topic.

**Figure 1:**
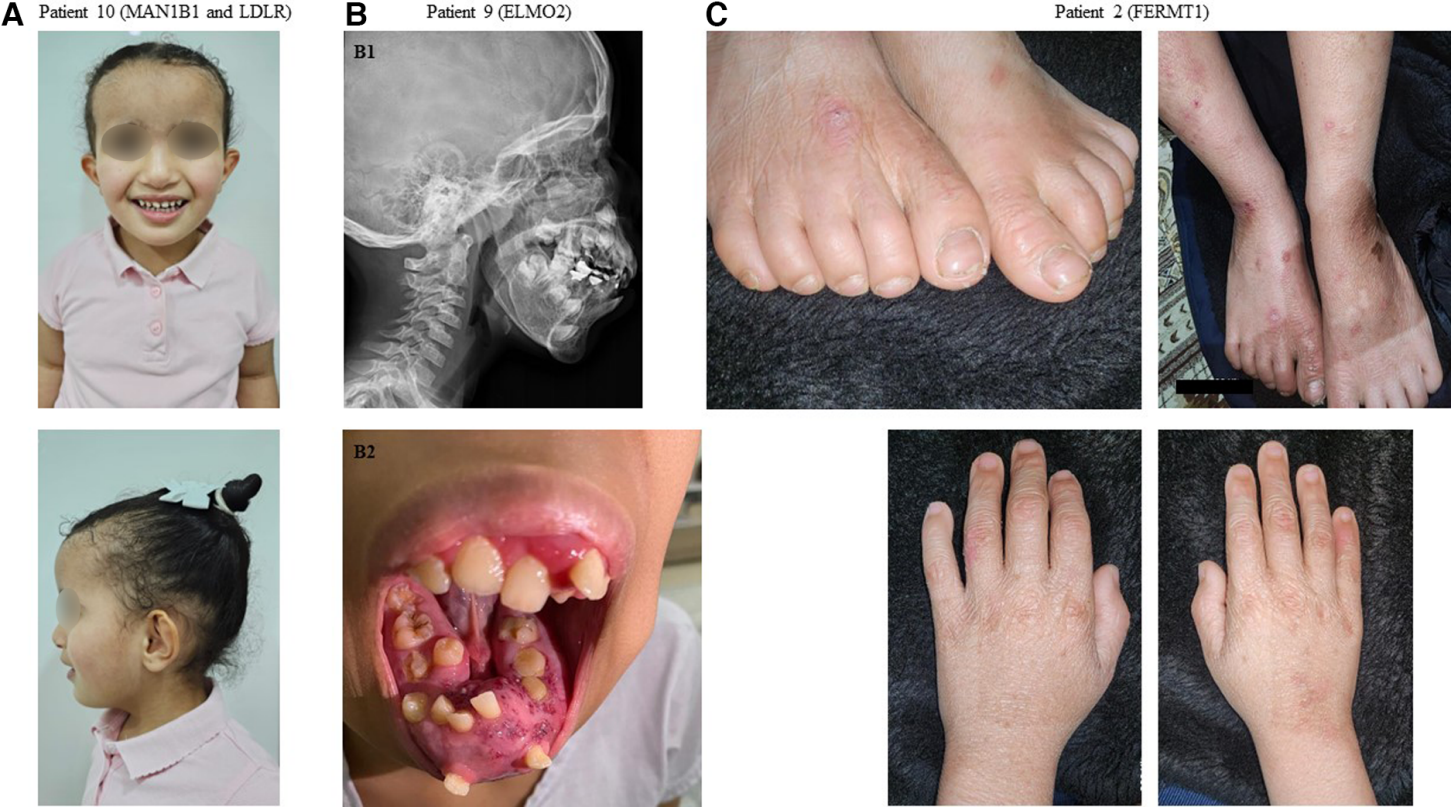
Representative clinical findings in patients with positive molecular diagnosis in our cohort. (**A**) Patient 10: Front and profile images demonstrating dysmorphic facial features and xanthomas over the antecubital fossa in a patient with a dual diagnosis of Rafiq syndrome (*MAN1B1* variant) and familial hypercholesterolemia (*LDLR* variant). (**B1**) Patient 9: A radiograph demonstrating mandibular hyperostosis in a patient with primary intraosseous vascular malformation (*ELMO2* variant). (**B2**) Patient 9: A clinical image demonstrating mandibular hyperostosis. (**C**) Patient 2: Images of the hands and feet demonstrating skin blistering and fragility in a patient with Kindler syndrome (*FERMT1* variant).

Interestingly in our study, two patients displayed dual molecular diagnosis, a phenomenon that has been well described in our highly consanguineous population (13). Patient 10 was found to have homozygous pathogenic *MAN1B1* and *LDLR* variants. These two variants fully explained her hybrid phenotype (Figure 1A) that comprised an *LDLR*-related aspect (elevated low-density lipoprotein and total cholesterol) and an *MAN1B1*-related aspect (macrocephaly, obesity, and global developmental delay). In the case of patient 6, however, the dual molecular diagnosis of homozygous pathogenic variants in *EPCAM* and *BEST1* did not correspond to a hybrid phenotype because her only presentation was diarrhea as part of *EPCAM*-related tufting enteropathy (OMIM# 613217). Ophthalmological evaluation is ongoing to evaluate for the presence of autosomal recessive *BEST1*-related Bestrophinopathy (OMIM# 611809).

The limitations of this study are that it contained only a small and selective single-center-based cohort with a relatively high consanguinity rate and uncertainty regarding *de novo* variants, because we implemented a proband-only (single) exome approach. The small sample size could be attributed to the limited number of general pediatricians working at the KFSH&RC. Of note, the KFSH&RC enabled a designated fund to offer patients CES at no cost to their families, which helped eliminate disparities based on financial constraints or insurance coverage (14).

## Conclusion

The findings of this study contribute evidence endorsing the ordering of CES by general pediatricians. We highlight how general pediatricians can effectively deploy CES to shorten the diagnostic odyssey of patients and improve overall patient care. Because of the demonstrably high clinical utility of CES, we hope that our results empower general pediatricians to advocate for expanded CES adoption.

## Data Availability

The original contributions presented in the study are included in the article/Supplementary Material, further inquiries can be directed to the corresponding author.
